# Effectiveness of thyme honey in the management of xerostomia in geriatric patients with end-stage renal disease: a randomized controlled clinical trial with a biochemical assessment

**DOI:** 10.1186/s40001-023-01351-9

**Published:** 2023-10-07

**Authors:** Suzan S. Ibrahim, Asmaa Abou-Bakr, Dalia M. Ghalwash, Radwa R. Hussein

**Affiliations:** 1 Oral Medicine and Periodontology, Faculty of Dentistry, Ain Shams University, Cairo, Egypt; 2 Faculty of Oral and Dental Medicine, Nahda University in Beni Seuf City, Beni Seuf, Egypt; 3 Oral Medicine and Periodontology, Faculty of Dentistry, The British University in Egypt, El Sherouk City, Egypt

**Keywords:** Thyme, Honey, ESRD, Geriatric, Xerostomia

## Abstract

**Background:**

Taking into consideration the value of the oral health condition in geriatric people with end-stage renal disease (ESRD) associated with xerostomia and believing that salivary stimulants or substitutes could potentially be used to manage this condition. This study aimed to evaluate the clinical effectiveness of thyme honey as oral rinse in geriatric patients with ESRD using the subjective dry mouth score as a primary objective and to assess the effect of thyme honey on the salivary nitric oxide level, salivary flow rate, and salivary ph in addition to objective dry mouth score as a secondary objective.

**Methods:**

This was a single blinded randomized controlled trial with two equal arms, the interventional arm (thyme honey oral rinse) and the control arm (saline). Twenty-eight geriatric patients with ESRD undergoing hemodialysis complained of xerostomia were recruited from the renal dialysis center. Patients in both arms followed the same administration protocol either with thyme honey oral rinse or saline. The following clinical parameters (the subjective and objective dry mouth scores, salivary flow rate, salivary ph, and salivary nitric oxide (NO) levels) were evaluated for both groups at different intervals (baseline, 1 week, and 1 month).

**Results:**

In the current study, it was found that both the subjective and objective dry mouth scores were significantly lower after one month of using thyme honey oral rinse (1.86 ± 0.66^B^) and (2.21 ± 0.43^B^) respectively, than the control group (3.07 ± 0.73^B^) and (3.07 ± 0.83^B^), respectively with a (*p* < 0.001). Also, the salivary flow rate was significantly higher after one month of using thyme honey oral rinse (1.56 ± 0.51^A^), than the control group (0.78 ± 0.27^A^) with a (*p* < 0.001). For the NO levels, there was a significant increase in measured value after 1 month in the intervention group (*p* < 0.001), while for the control group the change was not statistically significant (*p* = 0.166).

**Conclusions:**

The results of the current study have revealed the efficacy of Thyme honey oral rinse in the management of xerostomia in geriatric patients with ESRD.

*Trial registration* The ClinicalTrials.gov Identifier for this study is NCT05247008.

## Introduction

In the past two to three decades, extensive research has been conducted on the oral and dental health of elderly people. Dental caries, tooth loss, periodontitis, xerostomia, oral pre-cancerous or malignant lesions, and oral health-related quality of life were the oral conditions associated with older patients [1].

For the foreseeable future, an aging population with a high rate of coexisting illnesses is anticipated to exist with a prevalence of 46.3% chronic kidney disease (CKD) [2], and the number of patients affected by CKD has also been increasing, affecting an estimated 843.6 million individuals worldwide in 2017 [3], 66% hypertension, and 23% diabetes [4, 5], while the prevalence of ESKD grew by a median 43% [6]. CKD is becoming prevalent among the general population around the world [7], and the impact of ESRD on the world's health system is rising quickly [8]. Patients with ESRD tend to have xerostomia more frequently, with prevalence rates ranging from 28.2 to 78.8% [9].

Hemodialysis (HD) has a significant effect on salivary secretion and the biochemical composition of saliva, as patients on HD have decreased salivary flow rates [10]. A fluid-restricted diet, the use of several drugs induced xerostomia, the dialysis method itself, and/or salivary gland fibrosis and atrophy may all contribute to dry mouth in ESRD patients [11], additional factors like aging, hormonal imbalances, social and psychological issues could have an impact too [10, 12].

The subjective sense of oral dryness known as xerostomia is most usually associated with either diminished salivary flow or altered salivary composition. On the other side, Hyposalivation is objectively determined as a reduction in salivary flow rate. Xerostomia is a somewhat common condition, especially in elderly adults, and it can have serious effects on a person's overall health, including oral health [13].

In earlier investigations, a variety of pharmacological and non-pharmacological treatments based on stimulating salivary gland flow were evaluated to treat xerostomia and increase salivary flow in individuals with ESRD. Local stimulation as chewing of gum or citrus solid food or fruits, using various mouthwash formulas, low-level laser therapy, and acupuncture were used in the management of xerostomia, while, systemic stimulation with drugs like, angiotensin-converting enzyme inhibitors, cevimeline, pilocarpine, and angiotensin-receptor antagonists can all be used to mechanically stimulate salivary glands [14–18], but due to their cholinergic effect, these drugs have various contraindications like asthma, chronic obstructive pulmonary disease, heart failure, epilepsy, hyperthyroidism, glaucoma, gastric ulcer, kidney stones and Parkinson’s disease [19], and also due to their short relief duration, patients must take them for the rest of their lives [20].

Overall, it appears that the therapies that are currently accessible do not offer a long-term, complete, or successful management of xerostomia. This has increased the need for additional research into alternative therapies for the treatment of xerostomia [10], especially in elderly patients with various systemic diseases that may get unwanted side effects from the above-mentioned drugs.

Honey has many therapeutic potentials, and it doesn't cause negative effects like other pharmaceutical treatments [21, 22]. Thyme honey, a propolis gel product with potent antibacterial, antioxidant, antifungal, and immunomodulatory effects [23–27], is a novel alternative for the management of xerostomia [28]. Owing to the high sugar content of honey, it is thought that its presence in the mouth cavity has a sialagogue effect, causing the salivary glands to secrete more saliva [28, 29].

The production of saliva is significantly influenced by nitric oxide (NO), which is a biological messenger and a free radical [30]. NO is the primary molecule for signaling homeostasis and has a significant role in saliva secretion [31]. NO is a crucial biochemical marker engaged in the salivary glands' pathological and physiological functions. Increased oral mucus production and mucosal blood flow are indicated by high levels of nitrate and nitrite (stable metabolites of NO) in normal saliva [30–32].

Endogenous nitric oxide production in chronic renal failure patients is controversial. It has been suggested that the increase in the NO concentration may improve some pathological changes in uremia patients. The increase in NO in patients on hemodialysis could be due to hemodialysis membrane and/ or lack of renal excretion [33, 34]***. ***Blichard et al. [35] have stated that NO is an important biomarker in monitoring hemodialysis effects on salivary NO concentrations.

To the best of the author’s knowledge, there are no published data regarding the use of Thyme honey oral rinse in the management of xerostomia in geriatric patients with ESRD. So regarding the up mentioned properties of Thyme honey, we hypnotized that using thyme honey oral rinse could improve subjective and objective dry mouth scores by increasing the salivary NO levels.

## Materials and methods

### Sample size calculation

A power analysis was designed to have adequate power to apply a statistical test of the null hypothesis that there is no difference would be found between tested groups regarding the perception of xerostomia. By adopting alpha (*α*) and beta (*β*) levels of (0.2) (i.e., power = 95%), and a critical *z*-value of (1.96) calculated based on the results of a previous study^1^ the minimum required sample size (*n*) was found to be (28) cases (i.e., 14 cases per group). Sample size calculation was performed using G*Power version 3.1.9.7^2^According to a previous study by Yu et al. [36], summated xerostomia inventory (SXI) was 14.1 ± 5.8 at baseline, in comparison to 11.2 ± 4.6 after treatment.

### Study design

This study was designed as a single-blinded randomized controlled clinical trial with a biochemical assessment. Two arms (intervention and control) and with a 1:1 allocation ratio.

### Patient selection

Patients in both groups (intervention and control) were geriatric with ESRD undergoing hemodialysis who were complaining of xerostomia were selected from the hemodialysis center at Benha university hospital. Ethical approval of the study from the Faculty of Dentistry Ain Shams University Research Ethics Committee (FDASU-REC). The procedures were fully explained to the patients and they were asked to sign an informed consent.

### Randomization and masking

The patients were randomized from the beginning of the treatment to either the intervention or the control arm by implementing simple randomization using the envelope method. Based on this method, a pack of sealed envelopes including a card with either the word ‘intervention arm’ or ‘control arm’ written inside, was given to each patient after the agreement to participate in the study. Depending on which card was selected by the patients, they were allocated to the respective arm. The process of randomization and allocation of the participants to the two groups was overseen by an external, to study, third party.

The xerostomia was determined according to the following criteria:

1. Subjective symptoms of oral dryness. [37]
  Using a questionnaire was recorded according to the following:
  Q1. Does your mouth feel dry?
  Q2. Do you sip liquids to aid in swallowing dry food?
  Q3. Does your mouth feel dry when eating a meal?
  Q4. Does the amount of saliva in your mouth seem to be too little?
  Subjects who answered affirmatively to at least one of the questions related to oral dryness were considered positive for subjective complaints of oral dryness [37].
2. Objective dry mouth score. [38]
  The patients were examined for signs of the dry mouth include:
3. loss of pooled saliva.
4. Mouth mirror stickiness.
5. Stringy or foamy appearance.
6. Labial dehydration.
7. Irresponsiveness to parotid stimulation.

Objective dry mouth scores were calculated as the number of observed dry mouth signs (0–5), and patients with a score of less than 2 were excluded [39].

Before the recruitment, all patients were screened based on the following criteria:

- *Inclusion criteria* both genders, aged above 60 years, all patients were clinically diagnosed with ESRD undergoing hemodialysis, patients on hemodialysis** ≥ **3 months [12]***,*** all patients were complaining of xerostomia, objective dry mouth score from (2–5), subjective dry mouth score from (1–4), patients were able to make a reliable decision or communications.
- *Exclusion criteria* alcohol, smoking, patient who underwent a kidney transplant, patients with any autoimmune disease, malignancy, or diabetes mellitus [28]***,*** and patients with known hypersensitivity to thyme honey.

History was obtained and recorded from patients for screening of major risk factors of oral diseases. Personal history, demographic data (age, sex, marital status, occupation, education level). Dental chief complaint, medical and surgical history, including dental problems. Drug history, including currently prescribed drugs. History of renal disease, hemodialysis, frequency, and duration. Extra oral examination based on the information obtained from medical history, in addition to, cervical lymph nodes examination, TMJ examination, and salivary gland examination. Review of the medical files of patients including the related laboratory investigations (blood urea, serum creatinine, and hemoglobin level).

#### Treatment protocol (for the intervention group)

Thyme honey mouth rinse was prepared by the main investigator as (20 ml of thyme honey* diluted in 100 ml of purified water) [40] in opaque bottles that masked its content. It was used 3 times per day and patients were instructed not to swallow the thyme honey oral rinse.

#### The control group

Patients in the control arm followed the same protocol with normal saline rinses in the same opaque bottles 3 times per day.

Additionally, to the above-mentioned protocol patients in both arms were informed about [28]:

1. Necessary daily actions to combat xerostomia.
2. To determine and document any adverse effects or compliance caused by the thyme honey (as part of the safety of the intervention).
3. Patients were given detailed instructions on the best foods to eat and drinks to avoid because these things could make their xerostomia worsen.
4. Patients were instructed to provide the best treatment by emphasizing the value of regular oral health exams and good oral hygiene.

#### Treatment assessment evaluation for both groups

The treatment assessment of subjective dry mouth score, objective dry mouth score, salivary ph, and salivary flow rate were carried out at baseline, 2 weeks, and one month after starting the treatment protocol [28]***.*** Nitric oxide levels were measured at baseline and after 1 month of treatment [41].

After the treatment protocol period ends, all patients were followed up for 4 weeks of the treatment-free observation period. The evaluation of the treatment assessment was carried out according to the following parameters:

- Subjective dry mouth score.
- Objective dry mouth score.
- Salivary flow rate: Samples from patients (a day of dialysis visit) were collected between 8:00 AM and 11:00 AM to minimize the effects of the diurnal variability in salivary composition. Samples were collected before meals. During the time of collection, talking was prohibited, and unstimulated whole saliva was collected for 5 min by spitting method. The collection was timed, so that the flow rate (mL/min) was measured [42].
- Salivary pH: following saliva collection, pH was measured immediately using the narrow-range pH strip system (Merck). One drop of the collected saliva was placed on the test strip and its color change reflected the pH of the saliva.
- Salivary Nitric oxide: patients were also asked to collect the saliva using the spitting method in a sterile tube every 1 min for 5 min. The tubes were kept in a refrigerator (20 centigrade) before sending them to the laboratory to prevent changing the composition of the saliva [42].

*Salivary Nitric oxide levels* were determined by Nitric Oxide Assay Kit (Colorimetric) using Griess reaction: The Bio Diagnostic Nitrite Assay Kit provides an accurate and convenient method for measurement of endogenous nitrite concentration as an indicator of nitric oxide production in biological fluids. It depends on the addition of Griess Reagents which convert nitrite into a deep purple azo compound, photometric measurement of the absorbance due to this azo chromophore accurately determines NO_2_-concentration.

In an acid medium and in the presence of nitrite the formed nitrous acid diazotizes sulphanilamide and the product is coupled with N-(1–naphthyl) ethylene diamine. The resulting azo dye has a bright reddish-purple color which can be measured at 540 nm [43].

Saliva stored at − 20 °C to − 80 °C.

1-Standard sodium nitrite (50 µmol/L).

2-Sulphanilamide (10 µmol/L).

3-N (1 naphthyl) ethylene diamine (NEDA) (1 µmol/L).

The reagents are stable up to the expiry date specified when stored at + 4 to + 8 °C.

Mix well, and allow to stand for 5 min. Read absorbance of a sample (A sample) against sample blank and of standard (A standard) against standard blank at 540 nm (520–550 nm) Color stable for many hours. Linearity up to 200 µmol/L

$${\text{Nitrite}}\,{\text{oxide}}\,{\text{in sample }}{{\mu {\text{mol}}} \mathord{\left/ {\vphantom {{\mu {\text{mol}}} {\text{L}}}} \right. \kern-0pt} {\text{L}}} = {{({\text{A sample)}}} \mathord{\left/ {\vphantom {{({\text{A sample)}}} {({\text{A}}}}} \right. \kern-0pt} {({\text{A}}}}\,{\text{standard)}} \times {50}$$

The kit has been validated in saliva, and culture media. No sample purification from these sources is necessary other than some special instructions as described below. Store samples at – 20 ºC after collection. Antioxidants will interfere with the color development reaction. Azide, ascorbic acid, dithiothreitol, and mercaptoethanol will interfere with color development when present at a concentration as low as 100 μM. Alkyl amines, most sugars, lipids, or amino acids (except those containing thiol groups) do not interfere. Sensitivity When using the maximum amount of sample for the nitrite assay (100 μl), the detection limit is 2.5 μM.

### Statistical analysis

Categorical data were presented as frequencies and percentages. Numerical data were presented as mean and standard deviation values and were tested for normality using Shapiro–Wilk test. Parametric data were analyzed using independent *t*-test for intergroup comparisons and repeated measures ANOVA followed Bonferroni post hoc test for intragroup comparisons. While non-parametric data were analyzed using Mann–Whitney *U* test for intergroup comparisons and Friedman’s test followed by Nemenyi post hoc test for intragroup comparisons. Correlations were analyzed using Spearman’s rank order correlation coefficient. Associations were analyzed using independent *t*-test for parametric data and Mann–Whitney *U* test for non-parametric data. The significance level was set at *p* ≤ 0.05 for all tests. Statistical analysis was performed with R statistical software version 4.1.3 for Windows.^3^

## Results

The study was conducted on 28 geriatric patients with ESRD who were equally and randomly allocated to each of the tested groups (i.e., 14 cases each). There was no significant difference between both groups regarding sex (*p* = 0.695) and age (*p* = 0.646), intergroup comparisons and summary statistics for demographic data are presented in Table (1).

**Table 1:** Intergroup comparisons and summary statistics for demographic data

Parameter			Intervention	Control	*p*-value
Sex	Male	*n*	8	10	0.695
		%	57.1%	71.4%	
	Female	*n*	6	4	
		%	42.9%	28.6%	
Age (years)	Mean ± SD		69.43 ± 3.23	70.00 ± 3.28	0.646

There was no significant difference between both groups regarding having different medical conditions (*p* > 0.05), intergroup comparison, frequency and percentage values for medical condition are presented in Table (2).

**Table 2:** Intergroup comparison, frequency and percentage values for medical condition

Medical condition			Intervention	Control	*p*-value
Hypertension	No	*n*	3	1	0.280
		%	21.4%	7.1%	
	Yes	*n*	11	13	
		%	78.6%	92.9%	
Hypertension and chronic heart disease	No	*n*	11	14	0.067
		%	78.6%	100.0%	
	Yes	*n*	3	0	
		%	21.4%	0.0%	
Autoimmune nephritis	No	*n*	14	13	0.309ns
		%	100.0%	92.9%	
	Yes	*n*	0	1	
		%	0.0%	7.1%	

*ns* Non-significant

For serum creatinine and blood urea, the intervention group had significantly higher values than the control group (*p* < 0.05). For hemoglobin levels, the control group had significantly higher value than the intervention (*p* = 0.010). For the duration of hemodialysis, the difference was not statistically significant (*p* = 0.505). Intergroup comparison, mean and standard deviation values for medical parameters are presented in Table 3.

**Table 3:** Intergroup comparison, mean and standard deviation values for medical parameters

Parameter	Mean ± SD		*p*-value
	Intervention	Control	
Serum creatinine	10.29 ± 2.16	9.01 ± 0.59	0.042*
Blood urea	126.07 ± 20.30	110.01 ± 16.61	0.030*
Hemoglobin	8.46 ± 0.73	9.57 ± 1.30	0.010*
Duration of hemodialysis (months)	20.71 ± 8.84	23.00 ± 9.07	0.505

*Significant (*p* ≤ 0.05)

### Salivary parameters

#### A—subjective score

**Fig. 1.**
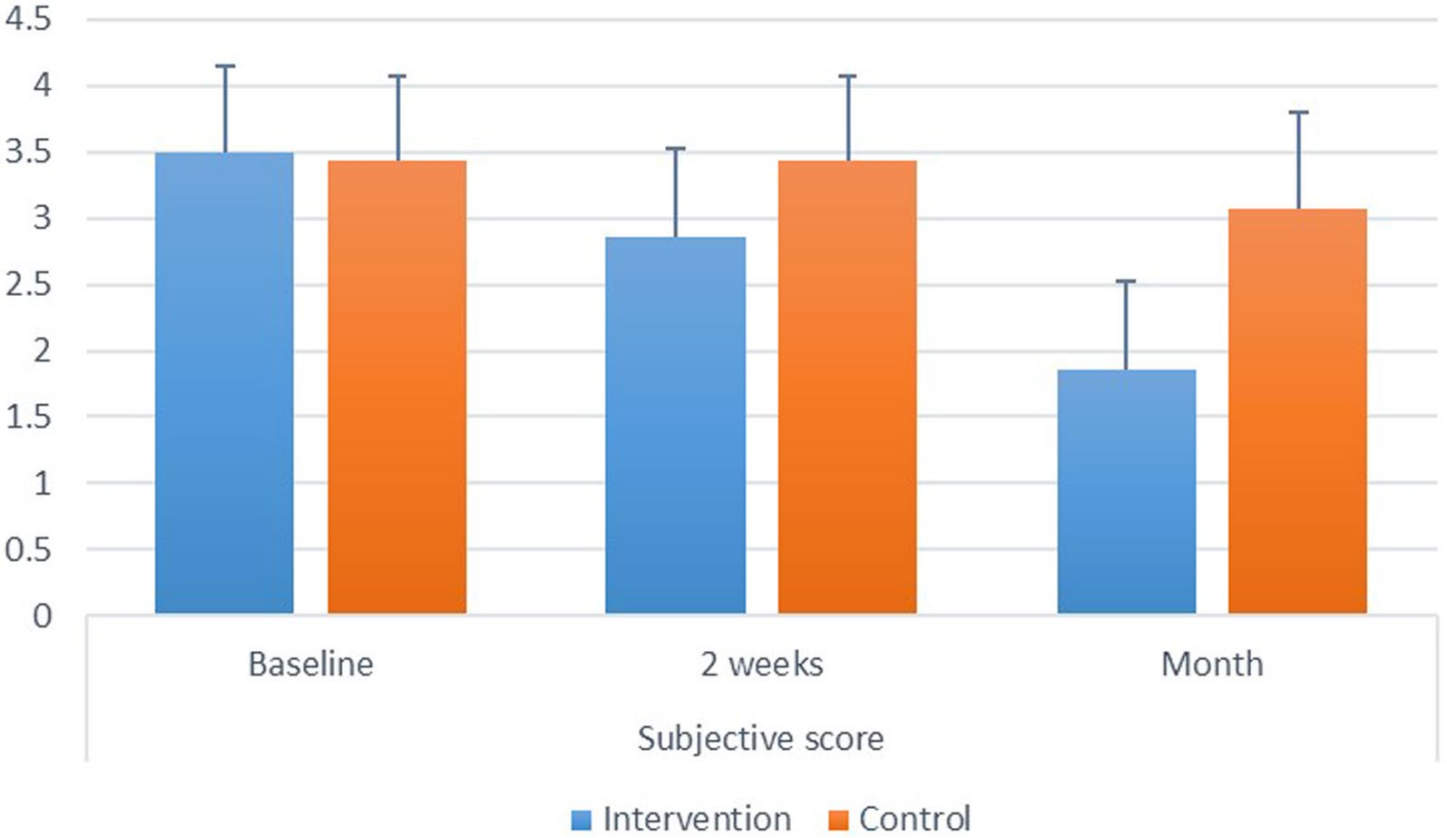
Showing intra and intergroup comparisons, mean and standard deviation values for subjective dry mouth score

*Intergroup comparisons* at baseline, there was no significant difference between both groups (*p* = 0.537). After 2 weeks and one month, control group had significantly higher scores than the intervention (*p* < 0.05). Intragroup comparisons: for both groups, there was a significant difference between values measured at different intervals, with value measured after 1 month being significantly lower than other intervals (*p* < 0.05). Inter and intragroup comparisons, mean and standard deviation values for subjective scores are presented in Fig. (1).

#### B—objective score

**Fig. 2.**
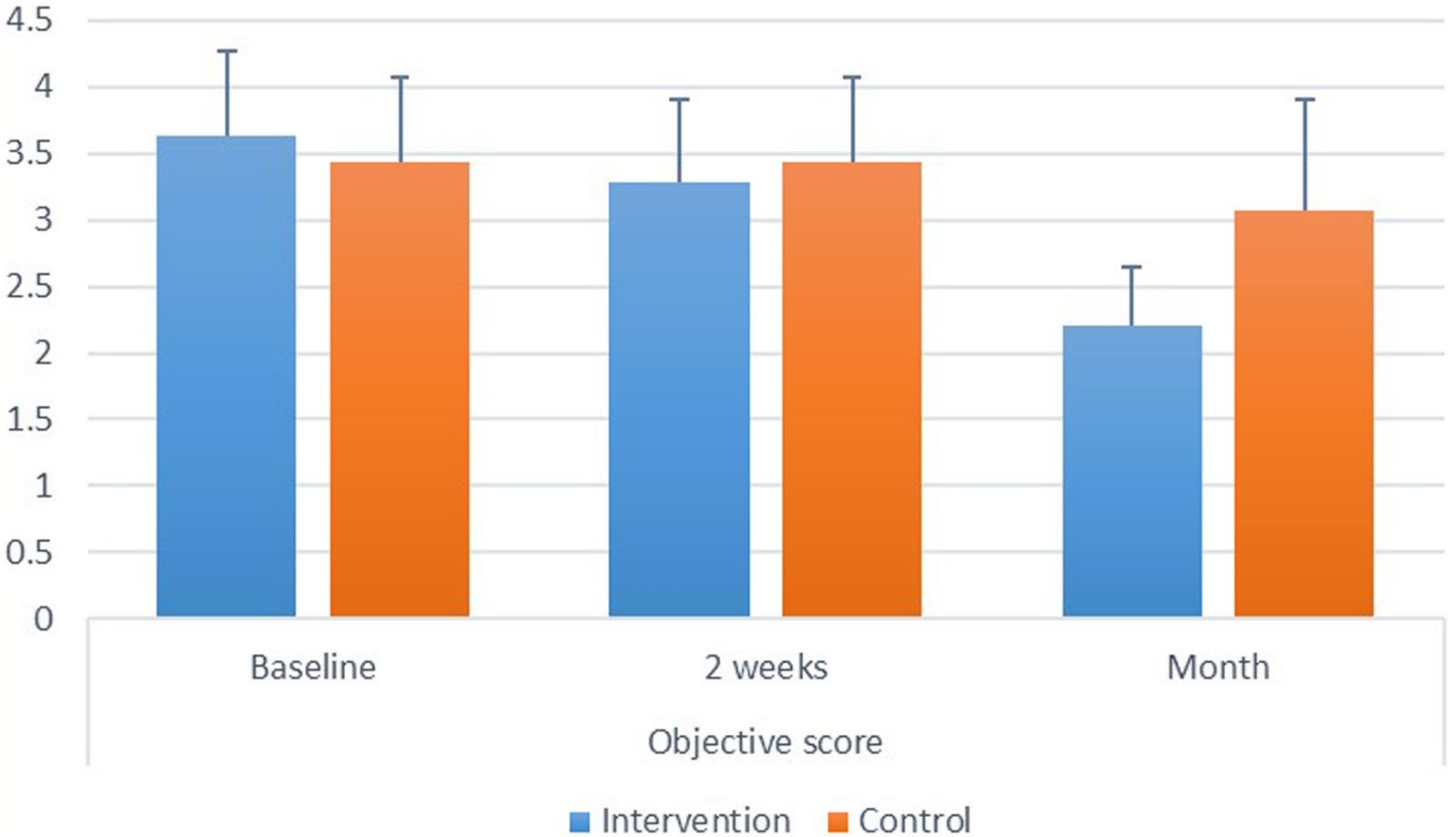
Showing inter and intragroup comparisons, mean and standard deviation values for objective dry mouth score

*Intergroup comparisons* at baseline and after 2 weeks, there was no significant difference between both groups (*p* > 0.05). After 1 month, control group had significantly higher scores than the intervention (*p* = 0.004). Intragroup comparisons: for both groups, there was a significant difference between values measured at different intervals, with value measured after 1 month being significantly lower than other intervals (*p* < 0.05). Inter and intragroup comparisons, mean and standard deviation values for objective scores are presented in Fig. (2).

#### C—salivary flow rate

**Fig. 3.**
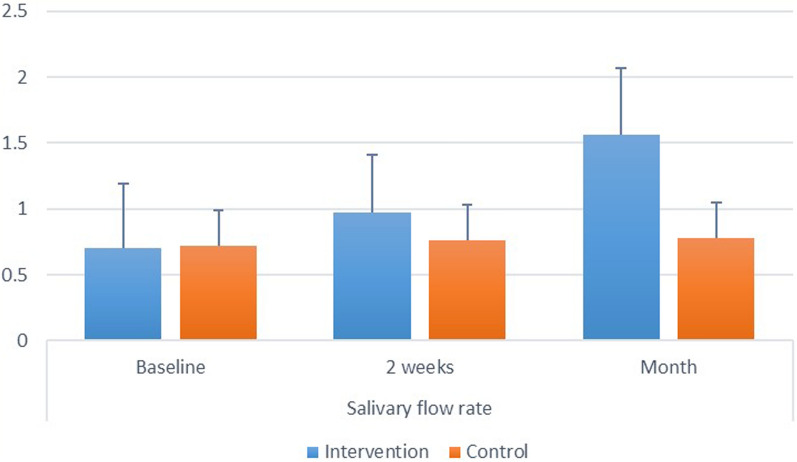
Showing inter and intragroup comparisons, mean and standard deviation values for salivary flow rate

*Intergroup comparisons* at baseline and after 2 weeks, there was no significant difference between both groups (*p* > 0.05). After 1 month, intervention group had significantly higher value than the control (*p* < 0.001). Intragroup comparisons: for the intervention, there was a significant difference between values measured at different intervals, with value measured after 1 month being significantly higher than other intervals (*p* < 0.001). While for the control group the difference was also significant but with value measured after 1 month being significantly higher than baseline value (*p* = 0.004). Inter and intragroup comparisons, mean and standard deviation values for salivary flow rate are presented in Fig. (3).

#### D—salivary pH

**Fig. 4.**
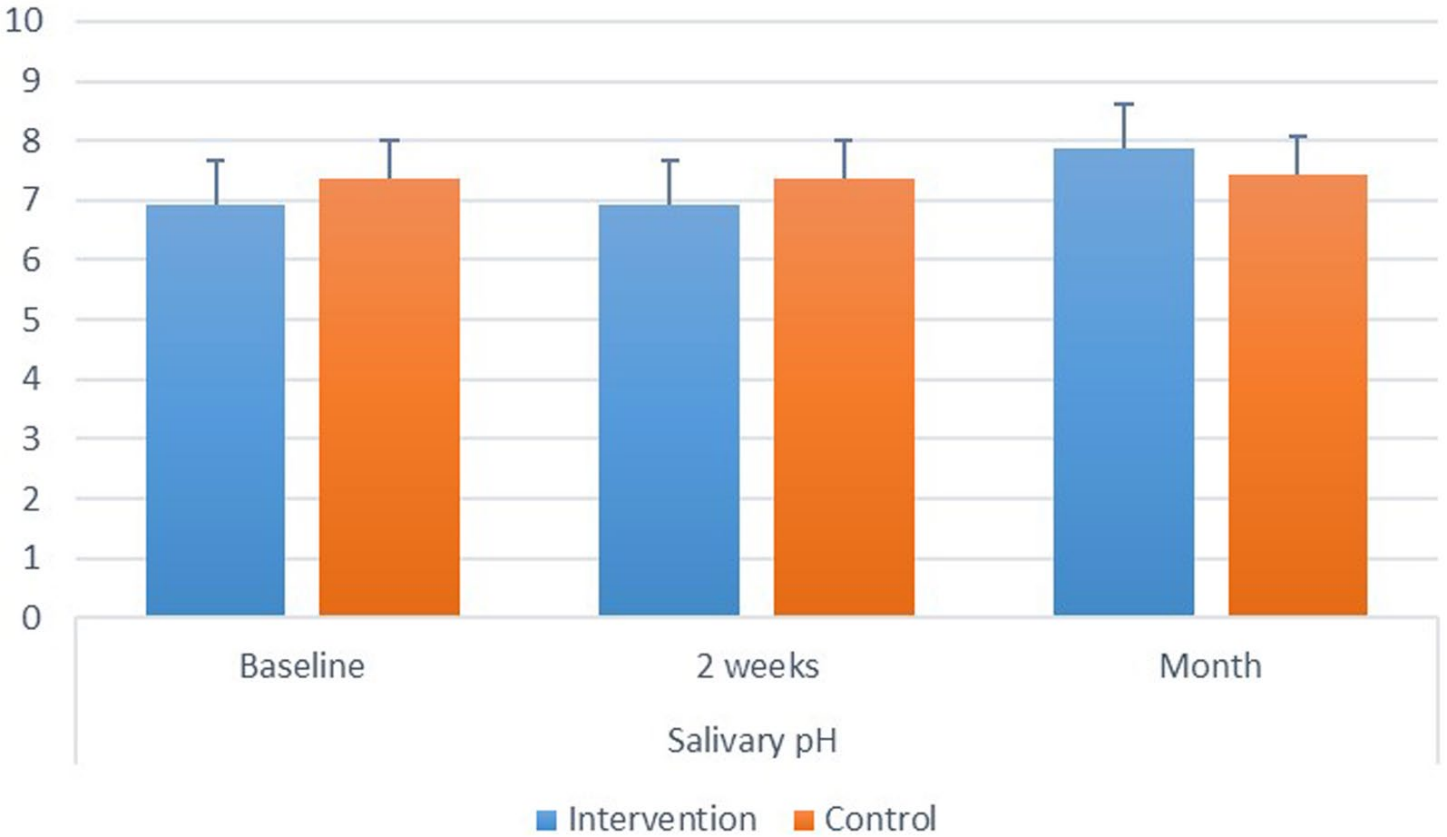
Showing inter and intragroup comparisons, mean and standard deviation values for salivary pH

Inter and intragroup comparisons, mean and standard deviation values for salivary pH are presented in Fig. (4).

*Intergroup comparisons* at all intervals, there was no significant difference between both groups (p > 0.05). Intragroup comparisons: for the intervention, there was a significant difference between values measured at different intervals, with value measured after 1 month being significantly higher than other intervals (*p *< 0.001). While for the control group the difference was not statistically significant (*p* = 0.368).

#### E- Nitric oxide level

**Fig. 5.**
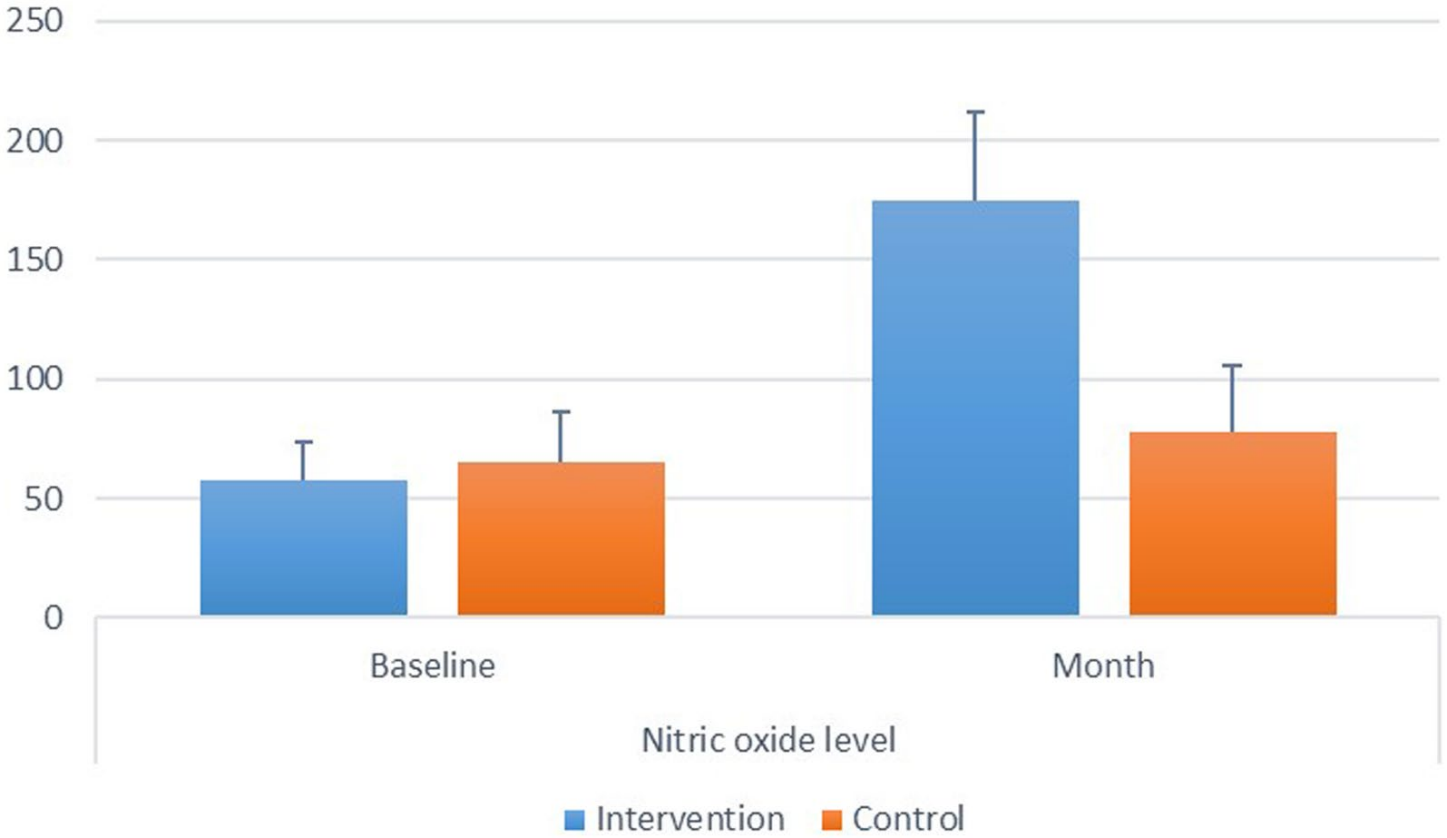
Showing inter and intragroup comparisons, mean and standard deviation values foe salivary nitric oxide levels

Inter and intragroup comparisons, mean and standard deviation values for nitric oxide level were presented in Fig. (5).

*Intergroup comparisons* at baseline, there was no significant difference between both groups (*p *= 0.270). However, after 1 month, the intervention had significantly higher value than the control group (*p* < 0.001). Intragroup comparisons: for the intervention, there was a significant increase in measured value after 1 month (*p* < 0.001). While for the control group the change was not statistically significant (*p* = 0.166).

## Discussion

Geriatric patients often have xerostomia, or dry mouth, which is frequently correlated with diminished salivary gland activity. The use of drugs, chronic illnesses including ESRD, and radiation therapy to the head and neck have all been implicated as causes of xerostomia in the elderly population [44]. Patients with ESRD on hemodialysis have a significantly low salivary flow rate, which is likely caused by the salivary gland’s atrophy and fibrosis for unknown causes [10]. Due to the various functions of saliva, patients suffering from chronic hyposalivation or xerostomia may experience problems in speech, taste, swallowing, and chewing as well as ill-fitting dentures and poor quality of life overall [45].

Hyposalivation also impairs immunological responses, lowers salivary pH and buffering capacity, and decreases oral defensive mechanisms. These signs and symptoms may raise the susceptibility to infectious oral disorders such as oral candidiasis, periodontitis, and cervical caries [39].

Nitric oxide (NO) may be essential for optimal salivary gland function and secretion, according to previous research [46]. The first recognized gas to function as a biological messenger is NO, a free radical. NO was first identified as a powerful vasodilator, but it was soon discovered to affect angiogenesis, function as a neurotransmitter, and be crucial to hosting defensive processes [46]. The primary contributor of salivary nitrate is the parotid gland; saliva contains almost three times as much nitrate as mixed whole saliva. Because it changes acinar cell calcium signaling in response to autonomic stimulation, NO is a key signaling molecule in controlling salivary secretion [47, 48].

In the current study, it was found that after 2 weeks and one month, the intervention group had significantly lower subjective dry mouth scores than control group (*p*<0.05), indicating a reduced perception of xerostomia and it was following previous study [28].

The objective dry mouth scores after 1 month, the intervention group had significantly lower scores than control group (*p*=0.004), indicating that the salivary flow rate has significantly increased in the intervention group as compared to the control one after one month (*p*<0.001), and this was further confirmed in our results by the significant increase of salivary flow rate after one month of using thyme honey mouth rinse, than the baseline, and that was in accordance with another study [28], which found that thyme honey was effective in stabilizing or reducing the degree of xerostomia in head and neck cancer patients, with gradual improvement over time.

Likewise, Lagerlof and Dawes [49], suggested that the topical application of honey leads to an increase in the salivary flow as honey has the ability to stimulate the gustatory system. Moreover, a recent study concluded that honey mouth care was effective in reducing the level of xerostomia [50].

In the present study, NO levels that were measured at 1 month for both groups, the intervention had significantly higher value than the control group (*p*<0.001), which was in accordance with previous studies [51, 52]. In addition to an earlier study reported that honey solution showed a tendency to increase total nitrite, a stable nitric oxide metabolite in different biological fluids from humans, including saliva, plasma, and urine [51]. This result is also in line with previous research that emphasized the healing power and the antibacterial action of honey through decreasing prostaglandin levels, elevating nitric oxide levels, and exerting prebiotic effects [52].

Afsaneh et al. [53] found that the salivary NO in the diabetic subjects with xerostomia was significantly lower than in diabetic subjects without xerostomia indicating that salivary nitric oxide level could monitor xerostomia in diabetic patients. Xia et al. [48] concluded that hypofunction of the salivary glands is associated with significant changes in nitrate and nitrite levels in the saliva and urine. Huskić J et al. [54] found that the salivary flow rate was significantly lower in patients with Parkinson’s disease than in healthy subjects, and salivary NO concentration was significantly lower than in healthy individuals.

The philosophy of utilizing thyme honey in xerostomia was founded on its’ salivary-stimulating properties [28]. Thyme honey contains several organic acids that could increase the flow of saliva and stimulate chemoreceptors in the oral cavity including ascorbic acid, malic acid, and citric acid [55]. Additionally, thyme honey unlike any other types of honeys has a high content of epicatechin gallate [56], which stimulates the neuroactive salivary secretomotor system thus increasing the salivary flow rate [57].

Regarding salivary pH in the intervention group, there was a significant difference between values measured at different intervals, with value measured after 1 month being significantly higher than other intervals (*p*<0.001). While for the control group the difference was not statistically significant (*p*=0.368). This indicating a significant rise in salivary pH over time while using thyme honey mouth rinse which was in accordance with a previous study [58], which reported that honey mouth rinse was significantly effective in increasing the salivary pH of the oral cavity.

This elevation in pH level could be due to a buffering capacity of thyme honey, salivary stimulation from thyme taste, and/or antibacterial activity against acid-producing bacteria. Since less bicarbonate is generated at low flow rates and pH drops, the salivary pH is largely reliant on the salivary flow rate [59]. The rise in salivary flow rate increases salivary buffering capacity which is vital for maintaining a pH level in saliva and plaque. Salivary stimulation increases the bicarbonate concentration in saliva [60], which raises the salivary pH, and considerably enhances its buffering capacity; therefore, saliva is much more effective in neutralizing and buffering acids arising in plaque from carbohydrate fermentation by microorganisms and food acids [61, 62].

The higher salivary pH in ESRD patients could be due to a higher ammonia concentration in saliva due to the hydrolysis of urea by the urease enzyme [63]. However, another study by Ghazali Norzalina et al. [64], who investigated the changes in salivary pH level, salivary flow rate, and salivary buffering capacity after consumption of tualang honey, and found a significant increase in the salivary flow rate after two weeks of treatment but reported a non-significant change in salivary pH.

Even though honey has an endogenous pH of (4.2), chewing it did not cause the pH to drop below the crucial level of 5.5 which is linked to enamel demineralization. Based on earlier findings showing that honey had antibacterial capabilities against medically significant bacteria, honey's antibacterial action against cariogenic bacteria may overcome its pH-reducing effects [65–67]. The pH of pure honey is approximately (3.9), making it acidic. In the absence of saliva, the solubility-reducing component in honey can activate. The micro-hardness of enamel is increased when honey is topically applied, thus preventing caries. Consequently, it has been hypothesized that honey is less cariogenic in people with the dry mouth [68].

According to the above-mentioned findings in the current study, thyme honey mouth rinse significantly reduced the symptoms and severity of xerostomia in the elderly patients with ESRD in the current study and this was in accordance with an earlier study by Charalambous et al. [28] who evaluated the effectiveness of thyme honey as a means for managing radiation-induced xerostomia and his findings supported the claim that the properties of thyme honey allow comprehensive and effective management of xerostomia in H&N cancer patients during and after radiotherapy.

## Conclusions

- Using thyme honey as an oral rinse proved to be very efficient in reducing subjective and objective dry mouth scores thus relieving xerostomia in geriatric patients with ESRD.
- Both salivary flow rate and salivary NO levels were significantly higher after one month of using thyme honey oral rinse than the control group without known negative side effects.
- Thyme honey would be a promising alternative to the currently used medications for treating xerostomia.

## Limitations

This study only involved one center; to further validate the results, a multi-centered study with a larger sample size must be conducted. Additionally, this study focused on geriatric patients with ESRD, while additional trials targeted different categories of patients with xerostomia to gauge their effectiveness across different demographics.

## Data Availability

The data that support the findings of this study are available from hemodialysis center in Egypt but restrictions apply to the availability of these data, which were used under license for the current study, and so are not publicly available. Data, however, available from the corresponding author upon reasonable request.

## Abbreviations

ESRD: End-stage renal disease
NO: Nitric oxide
CKD: Chronic kidney disease
HD: Hemodialysis

## Footnotes

1 Charalambous, Andreas, et al. "The effectiveness of thyme honey for the management of treatment-induced xerostomia in head and neck cancer patients: A feasibility randomized control trial." European Journal of Oncology Nursing 27 (2017): 1–8.

2 Faul, Franz, et al. "G* Power 3: A flexible statistical power analysis program for the social, behavioral, and biomedical sciences." *Behavior research methods* 39.2 (2007): 175–191.

3 R Core Team (2022). R: A language and environment for statistical computing. R Foundation for Statistical Computing, Vienna, Austria. URL https://www.R-project.org/.

## References

[1] Murray-Thomson W (2014). Epidemiology of oral health conditions in older people. Gerodontology.

[2] Levey AS (2009). A new equation to estimate glomerular filtration rate. Ann Intern Med.

[3] Jager KJ, Kovesdy C, Langham R (2019). A single number for advocacy and communication-worldwide more than 850 million individuals have kidney diseases. Kidney Int.

[4] Centers for Disease Control and Prevention. National diabetes fact sheet: general information and national estimates on diabetes in the United States, 2007. U.S. Department of Health and Human Services, Centers for Disease Control and Prevention. Atlanta, GA: 2008.

[5] Cutler JA (2008). Trends in hypertension prevalence, awareness, treatment, and control rates in United States adults between 1988–1994 and 1999–2004. Hypertension.

[6] United States Renal Data System. 2018 USRDS annual data report: epidemiology of kidney disease in the United States. Bethesda, MD: National Institutes of Health, National Institute of Diabetes and Digestive and Kidney Diseases; 2018.

[7] Lv JC, Zhang LX (2019). Prevalence and disease burden of chronic kidney disease renal fibrosis: mechanisms and therapies. Adv Experim Med Biol.

[8] Thurlow JS, Joshi M, Yan G, Norris KC, Agodoa LY, Yuan CM, Nee R (2021). Global epidemiology of end-stage kidney disease and disparities in kidney replacement therapy. Am J Nephrol.

[9] Stevens LA, Viswanathan G, Weiner DE (2010). CKD and ESRD in the elderly: current prevalence, future projections, and clinical significance. Adv Chronic Kidney Dis.

[10] Bossola M (2019). Xerostomia in patients on chronic hemodialysis: an update. Semin Dial.

[11] Kumar T, Kishore J, Kumari M, Rai A, Rai S, Jha A (2020). Evaluation of salivary flow rate, pH, and buffer capacities in end-stage renal disease patients versus control—a prospective comparative study. J Family Med Prim Care.

[12] Bots CP, Brand HS, Veerman ECI, Korevaar JC, Valentijn-Benz M, Bezemer PD, Valentijn RM, Vos PF, Bijlsma JA, Wee PM, Van Amerongen BM, Amerongen AVN (2005). Chewing gum and a saliva substitute alleviate thirst and xerostomia in patients on haemodialysis. Nephrol Dial Transplant.

[13] Villa A, Wolff A, Aframian D, Vissink A, Ekström J, Proctor G, McGowan R, Narayana N, Aliko A, Sia YW, Joshi RK, Jensen SB, Kerr AR, Dawes C (2015). Pedersen AM (2015) World workshop on Oral medicine VI: a systematic review of medication-induced salivary gland dysfunction: prevalence, diagnosis, and treatment. Clin Oral Investig.

[14] Yang LY, Yates P, Chin CC, Kao TK (2010). Effect of acupressure on thirst in hemodialysis patients. Kidney Blood Press Res.

[15] Jagodzińska M, Zimmer-Nowicka J, Nowicki M (2011). Three months of regular gum chewing neither alleviates xerostomia nor re- duces overhydration in chronic hemodialysis patients. J Ren Nutr.

[16] Duruk N, Eser I (2016). The null effect of chewing gum during hemodialysis on dry mouth. Clin Nurse Spec.

[17] Yang G, Lin S, Wu Y (2017). Auricular acupressure helps alleviate xerostomia in maintenance hemodialysis patients: a pilot study. J Altern Complement Med.

[18] Yang LY, Chen HM, Su YC, Chin CC (2019). The effect of transcutane- ous electrical nerve stimulation on increasing salivary flow rate in hemodialysis patients. Oral Dis.

[19] Vives-Soler A, López-López J, Jané-Salas E (2017). Xerostomía y radioterapia de cabeza y cuello: actualización. Rev Colomb Cancerol.

[20] Pereira MSS, Silva BO, Santos FR (2015). Acupuntura: terapia alternativa, integrativa e complementar na Odontologia. Rev CROMG.

[21] Alam F, Islam MA, Gan SH, Khalil MI (2014). Honey: a potential therapeutic agent for managing diabetic wounds. Evid Based Complement Alternat Med.

[22] Belcher J (2014). Dressings and healing with honey. Br J Nurs.

[23] Yang C, Gong G, Jin E, Han X, Zhuo Y, Yang S, Song B, Zhang Y, Piao C (2019). Topical application of honey in the management of chemo/radiotherapy-induced oral mucositis: a systematic review and network meta-analysis. Int J Nurs Stud.

[24] Lima ICGDS, Fátima SoutoMaior L, Gueiros LAM, Leão JC, Higino JS, Carvalho AAT (2021). Clinical applicability of natural products for prevention and treatment of oral mucositis: a systematic review and meta-analysis. Clin Oral Investig.

[25] Charalambous M, Raftopoulos V, Paikousis L, Katodritis N, Lambrinou E, Vomvas D, Georgiou M, Charalambous A (2018). The effect of the use of thyme honey in minimizing radiation—induced oral mucositis in head and neck cancer patients: a randomized controlled trial. Eur J Oncol Nurs.

[26] Ramsay EI, Rao S, Madathil L, Hegde SK, Baliga-Rao MP, George T, Baliga MS (2019). Honey in oral health and care: a mini review. J Oral Biosci.

[27] Münstedt K, Momm F, Hübner J (2019). Honey in the management of side effects of radiotherapy—or radio/chemotherapy-induced oral mucositis. a systematic review. Complement Ther Clin Pract.

[28] Charalambous A, Lambrinou E, Katodritis N, Vomvas D, Raftopoulos V, Georgiou M, Paikousis L, Charalambous M (2017). The effectiveness of thyme honey for the management of treatment-induced xerostomia in head and neck cancer patients: a feasibility randomized control trial. Eur J Oncol Nurs.

[29] Barbieri T, Claudia K (2020). Current alternatives in the prevention and treatment of xerostomia in cancer therapy RGO. Rev Gaúch Odontol.

[30] Shaalan A, Carpenter G, Proctor G (2018). Inducible nitric oxide synthase-mediated injury in a mouse model of acute salivary gland dysfunction. Nitric Oxide.

[31] Hezel M, Weitzberg E (2015). The oral microbiome and nitric oxide homoeostasis. Oral Dis.

[32] Breseghelo ML, Guillo LA, Nogueira TE, Leles CR (2016). Nitric oxide concentration and other salivary changes after insertion of new complete dentures in edentulous subjects. Int J Dent..

[33] Matavulj A, Kovačević P, Huskić J, Veljković S, Rajkovača Z, Ponorac N (2008). Effects of haemodialysis and continous ambulatory peritoneal dialysis on nitric oxide serum concentration in patients with chronic renal failure. Acta Med Sal.

[34] Vucijak-Grgurevic M, Zvizdic F, Durak-Nalbantic A, Jahic E, Resic N, Huskic J (2018). Significance of nitric oxyde saliva concentration of the patients with renal failure on hemodialysis. Mater Sociomed.

[35] Blicharz TM, Rissin DM, Bowen M, Hazman RB, Di Cesare C, Bhatia JS (2008). Use of colorimetric test stripts for monitoring the effects of hemodialysis on salivary nitrite and uric acid in patients with end-stage renal disease: a proof of principle. Clin chem.

[36] Yu C, Tsaia YF, Fang JT, Yehf MM, Fang JY, Liu CY (2016). Effects of mouthwash interventions on xerostomia and unstimulated whole saliva flow rate among hemodialysis patients: a randomized controlled study. Int J Nurs Stud.

[37] Bardow A, Nyvad B, Nauntofte B (2001). Relationships between medication intake, complaints of dry mouth, salivary flow rate and composition, and the rate of tooth demineralization in situ. Arch Oral Biol.

[38] Osailan S, Pramanik R, Shirodaria S, Challacombe SJ, Proctor GB (2011). Investigating the relationship between hyposalivation and mucosal wetness. Oral Dis.

[39] Dalodom S, Lam-ubol A, Jeanmaneechotechai A, Takamfoo L, Intachai W, Duangchada K, Hongsachum B, Kanjanatiwat P, Vacharotayangul P, Trachootham D (2016). Influence of oral moisturizing jelly as a saliva substitute for the relief of xerostomia in elderly patients with hypertension and diabetes mellitus. Geriatr Nurs.

[40] Biswal BM, Zakaria A, Ahmad NM (2003). Topical application of honey in the management of radiation mucositis: a preliminary study. Support Care Cancer.

[41] Abadi PA, Koopaie M, Montazeri R (2020). Comparison of salivary nitric oxide and oral health in diabetic patients with and without xerostomia. Diabetes Metab Syndr.

[42] Navazesh M, Kumar SK (2008). Measuring salivary flow challenging and oppourtunities. J Am Dent Assoc.

[43] Montgomery HAC, Dymock J (1961). The determination of nitrite in water. Analyst.

[44] Ouanounou A (2016). Xerostomia in the geriatric patient: causes, oral manifestations and treatment. Compend Contin Educ Dent.

[45] Cho EP, Hwang SJ, Clovis JB, Lee TY, Paik DI, Hwang YS (2012). Enhancing the quality of life in elderly women through a programme to improve the condition of salivary hypofunction. Gerodontology.

[46] Rosignoli F, Goren NB, Leiros CP (2001). Alterations in nitric oxide synthase activity and expression in submandibular glands of NOD mice. Clin Immunol.

[47] Looms DK (2001). Nitric oxide and cGMP activate Ca2+-release processes in rat parotid acinar cells. Biochem J.

[48] Xia D, Deng D, Wang S (2003). Alterations of nitrate and nitrite content in saliva, serum, and urine in patients with salivary dysfunction. J Oral Pathol Med.

[49] Lagerlof F, Dawes C (1984). The volume of saliva in the mouth before and after € swallowing. J Dent Res.

[50] Das S, Mohanty S, Debnath S (2020). Effect of honey mouth-care on xerostomia among semiconscious and unconscious patients. Int J Res Pharm Sci.

[51] Al-Waili NS, Boni NS (2004). Honey increased saliva, plasma, and urine content of total nitrite concentrations in normal individuals. J Med Food.

[52] Al-Waili NS, Salom K, Butler G, Al AA (2011). Honey and microbial infections: a review supporting the use of honey for microbial control. J Med Food Oct.

[53] Afsaneh Abadi P, Koopaie M, Montazeri R (2020). Comparison of salivary nitric oxide and oral health in diabetic patients with and without xerostomia. Diabetes Metab Syndr.

[54] Huskić J, Paperniku A, Husić A, Alendar F, Mulabegović N (2005). Significantly reduced salivary nitric oxide synthesis in patients with Parkinson's disease. Bosn J Basic Med Sci.

[55] Davies AN, Davies AN, Epstein JB (2010). Salivary gland dysfunction. Oral complications of cancer and its management.

[56] Imtara H (2021). Chemical composition and antioxidant content of Thymus vulgaris honey and Origanum vulgare essential oil; their effect on carbon tetrachloride-induced toxicity. Veterinary World.

[57] Saito K, Mori S, Date F, Hong G (2015). Epigallocatechin gallate stimulates the neuroreactive salivary secretomotor system in autoimmune sialadenitis of MRL-Fas(lpr) mice via activation of cAMP-dependent protein kinase A and inactivation of nuclear factor κB. Autoimmunity.

[58] Ali A, Farooq L, Mahmood A, Ahmed SN, Ahmed A, Mujahid S (2021). Comparative evaluation of salivary ph with honey and vinegar mouth rinse in diabetic and healthy adults. J Pharma Res Int.

[59] Humphrey SP, Williamson RT (2001). A review of saliva: normal composition, flow, and function. J Prosthet Dent.

[60] Tamimi Iman A, Muhammad Q (2012). Effect of thymus vulgaris extract on streptococci and mutans streptococci, in comparison to chlorhexidine gluconate in vivo study. J Baghdad College Dentistry.

[61] Bardow A, Moe D, Nyvad B, Nauntofte B (2000). The buffer capacity and buffer systems of human whole saliva measured without loss of CO2. Arch Oral Biol.

[62] Rantonen, Panu. Salivary flow and composition in healthy and diseased adults. 2003.

[63] Hamid MJ, Dummer CD, Pinto LS (2006). Systemic conditions, oral findings and dental management of chronic renal failure patients: general considerations and case report. Braz Dent J.

[64] Ghazali N, Mohammed N, Ramli H, Yazid F, Ibrahim AZ (2019). Level of salivary flow rate, pH level, buffering capacity and after consumption of Malaysian Tualang honey: a preliminary study. J Int Dental Med Res.

[65] Digrak M, Yilmaz O, Ozcelik S (1995). In vitro antimicrobial effect of propolis collected in Elazig region. Turk J Biol.

[66] Steinberg D, Kaine G, Gedalia I (1996). Antibacterial effect of propolis and honey on oral bacteria. Am J Dentistry.

[67] Atwa AD, AbuShahba RY, Mostafa M, Hashem MI (2014). Effect of honey in preventing gingivitis and dental caries in patients undergoing orthodontic treatment. Saudi Dent J.

[68] Sela MO, Shapira L, Grizim I, Lewinstein I, Steinberg D, Gedalia I, Grobler SR (1998). Effects of honey consumption on enamel microhardness in normal versus xerostomic patients. J Oral Rehabil.

